# High resolution atomic force and Kelvin probe force microscopy image data of InAs(001) surface using frequency modulation method

**DOI:** 10.1016/j.dib.2020.105177

**Published:** 2020-01-25

**Authors:** Young Min Park, Joon Sik Park, Choong-Heui Chung, Sangyeob Lee

**Affiliations:** a Surface Technology Group, Korea Institute of Industrial Technology (KITECH), Incheon, 21999, Republic of Korea; b Department of Materials Science and Engineering, Hanbat National University, Daejeon, 34158, Republic of Korea

**Keywords:** InAs(001) surface, Frequency-modulation mode atomic force microscopy, Frequency-modulation mode Kelvin probe force microscopy, Semiconductor surface

## Abstract

This article provides data on the scanning tunnelling microscopy (STM), atomic force microscopy (AFM) and Kelvin probe force microscopy (KPFM) images of InAs(001) surface. Using the frequency-modulation (FM) method in AFM and KPFM, atomic resolution topography and contact potential difference (CPD) images of InAs(001) surface were obtained. The InAs(001) surface reconstruction images observed by STM and AFM are compared. The effect of AFM tip condition and tip-sample distance to AFM and KPFM imaging is verified by measuring frequency shift vs. tip-sample distance spectroscopy. This data article is related to the article entitled, “Kelvin prove force microscopy and its application” (Melitz et al., 2011) [1].

**Table:** Specifications Table

Subject	Materials Science
Specific subject area	Semiconductor surface, atomic force microscopy, Kelvin probe force microscopy
Type of data	Images (AFM, KPFM, STM)
How data were acquired	AFM (Omicron VT-AFM/STM) KPFM (Omicron VT-AFM/STM equipped with contact potential measurement system) STM (Omicron VT-STM)
Data format	Raw and analyzed
Parameters for data collection	AFM, KFPM scanning condition including conducting tip surface state and frequency shift according to tip-sample distance
Description of data collection	InAs(001) surface reconstruction was observed using an atomic resolution AFM operated in FM mode. Atomic resolution topography and CPD of InAs(001) surface were observed simultaneously using FM mode KPFM. Frequency shift vs. tip-sample distance spectroscopic data were collected on different positions on InAs(001) surface.
Data source location	Hanbat National University, Daejeon 34158, Republic of Korea Department of Chemistry and Biochemistry, University of California, San Diego, La Jolla, CA 92093, USA
Data accessibility	Raw data related to Fig. 2(c) and (d) are available within this article as a supplementary file. AFM, STM, and KPFM images are included in the article.
Related research article	Author's name: Wilhelm Melitz et al. Title: Kelvin probe force microscopy and its application Journal: Surface Science Reports https://doi.org/10.1016/j.surfrep.2010.10.001

**Table:** 

**Value of the Data** • FM mode AFM can be used to image the semiconductor surface with atomic resolution. FM mode KPFM can be applied to image local potential distribution and morphology of semiconductor surface simultaneously. • Detailed information of FM mode AFM and KPFM can provide a technical knowledge to acquire atomic resolution AFM and KPFM images. • The high resolution AFM and STM image of InAs(001) surface can be useful to the researchers who may study the surface reconstruction on InAs(001). The data on tip-sample distance vs. oscillation frequency shift should be useful to researchers who may analyse the surface potential of InAs(001).

## 1 Data description

**Fig. 1:**
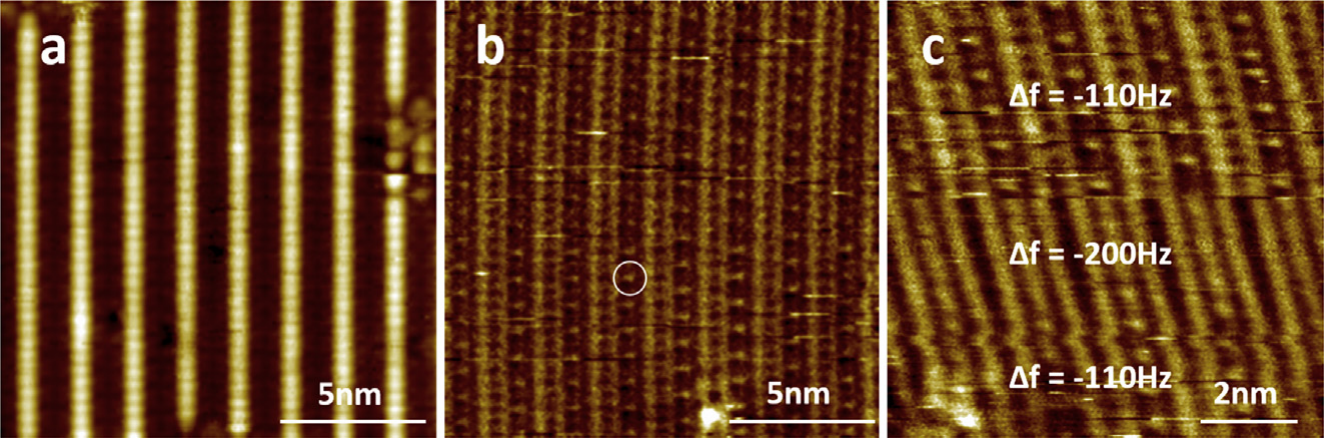
(a) 15 nm × 15 nm STM, (b) 15 nm × 15 nm and (c) 9 nm × 9 nm AFM images of InAs(001) surface. In (c), AFM tip was brought close to the sample surface by changing the frequency shift (set - free oscillation frequency) to −200 Hz.

**Fig. 2:**
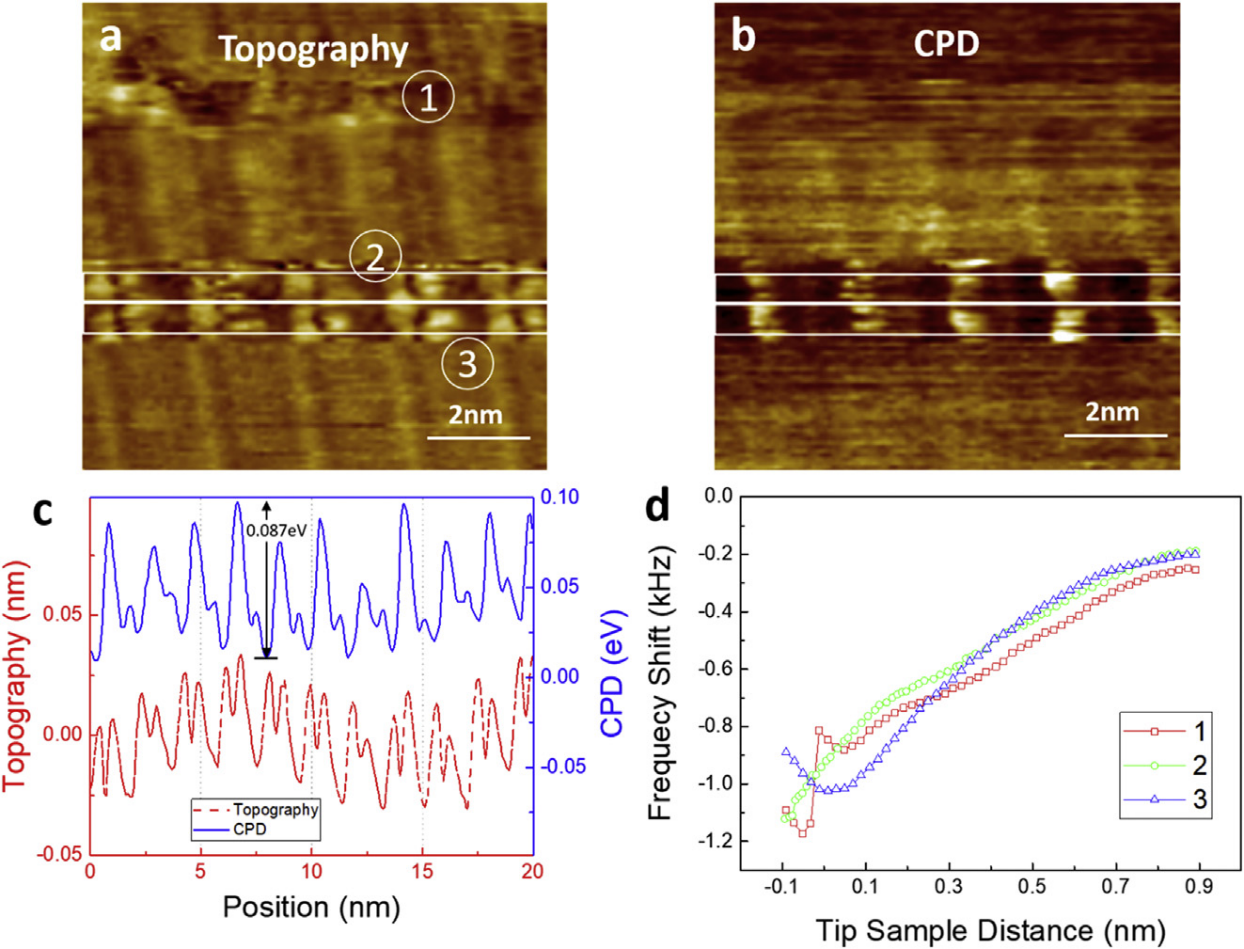
KPFM measurement of InAs(001) surface showing simultaneous (a) topography and (b) CPD. Averaged topography (CPD) line profiles, red dashed line (blue solid line), across white line box in the (a) and (b) are shown in (c). Frequency shift vs. tip-sample distance spectroscopy are measured in 1, 2, and 3 denoted in (a) and the data are shown in (d).

This data article provides STM, AFM, and KPFM images of InAs(001)-(4 × 2) reconstructed surface grown by MBE technique. Fig. 1 shows 15 nm × 15 nm (a) STM, (b) AFM, and (c) 9 nm × 9 nm AFM images of InAs(001)-(4 × 2) surface reconstruction. As shown in the STM image of Fig. 1(a), InAs(001) surface has a bright row and dark trough structure. The detailed information of surface reconstruction can be found elsewhere [1,2]. The AFM image of Fig. 1(b) also shows the row and trough structure. However, the AFM image shows a bright feature in the trough region as denoted by a circle in Fig. 1(b). When the AFM tip is brought close to sample surface while scanning by adjusting the frequency shift, the bright feature become unclear and cannot be distinguished as shown in Fig. 1(c). Fig. 2 shows KPFM measurements of (a) topography and (b) CPD of InAs(001) surface. The correlation between surface potential and structure can clearly be seen in the simultaneous topography and CPD line profiles in Fig. 2(c). The row and trough structures are observed in the white line box denoted in Fig. 2(a). The potential difference between the bright row and dark trough ranges from 0.038 to 0.087 eV. The topography and CPD images become clear in the white line box during scanning. The frequency shift spectroscopy was measured at 3 points as denoted in Fig. 2(a) and the spectroscopic data are shown in Fig. 2(d). During scanning, the surface ad-atom can be attached to the AFM tip, which results in the different spectroscopic behavior as shown in Fig. 2(d). The attached ad-atom altered the atomic structure and surface potential at the end of AFM tip, which result in the different topography and CPD images as shown in the white line box of Fig. 2(a) and (b).

## 2 Experimental design, materials, and methods

All experiments were performed in an ultrahigh vacuum chamber with a base pressure of 2 × 10^−10^ Torr. The InAs samples are commercially available InAs wafer (Wafer Tech) with a 200 nm thick InAs surface layer grown by MBE. The STM and AFM measurements were performed with Omicron VT-AFM/STM system with an Omicron KPFM module. In order to obtain high resolution, AFM and KPFM imaging were performed in FM mode using super sharp silicon AFM tip (Nanosensors). The Si AFM tip was coated with 2–3 nm of Cr to make the tip conductive for KPFM imaging. FM mode KPFM was performed with a cantilever with resonant frequency of approximately 270 kHz.
