# Supplementary material for: High resolution atomic force and Kelvin probe force microscopy image data of InAs(001) surface using frequency modulation method
Source: Data Brief. 2020 Jan 25;29:105177. doi: 10.1016/j.dib.2020.105177 (PMC7005429; doi:10.1016/j.dib.2020.105177)
Supplement: Multimedia component 2 [file mmc2.pdf]

X1 (Position, nm)	Y1 (CPD, eV)	X2 (Position, nm)	Y2 (Topography, nm)
0.00747	-0.02209	0.01009	0.01451
0.06726	-0.01992	0.08487	0.01346
0.11211	-0.01677	0.1388	0.01077
0.15695	-0.01233	0.2106	0.0095
0.20179	-0.00799	0.32718	0.00982
0.24664	-0.00385	0.40778	0.01251
0.28401	9.86E-05	0.45249	0.01568
0.35874	0.00276	0.47028	0.01916
0.44096	0.00385	0.50595	0.02407
0.50075	0.00178	0.55051	0.03072
0.5157	-0.00247	0.54135	0.03515
0.54559	-0.00602	0.58599	0.0399
0.55734	-0.01052	0.60377	0.0437
0.58937	-0.01416	0.6036	0.04782
0.57655	-0.01746	0.63028	0.05304
0.60858	-0.02202	0.63909	0.05684
0.64702	-0.02447	0.66583	0.06064
0.72389	-0.02506	0.6925	0.06618
0.7303	-0.02008	0.66539	0.07109
0.76233	-0.01627	0.73696	0.07537
0.77514	-0.01247	0.7368	0.07901
0.79436	-0.009	0.76354	0.08297
0.8328	-0.00478	0.83516	0.08597
0.85842	-0.00165	0.89807	0.08297
0.88405	0.00165	0.95208	0.07822
0.90327	0.00503	0.99713	0.07331
0.98014	0.00672	1.0063	0.06872
1.05061	0.00655	1.06924	0.06492
1.10186	0.00376	1.12328	0.05954
1.19154	-5.49E-04	1.14144	0.05416
1.25561	-0.0052	1.20445	0.04878
1.30685	-0.00866	1.25852	0.04276
1.3581	-0.01255	1.3036	0.03722
1.39013	-0.01509	1.35759	0.03295
1.42857	-0.01805	1.39366	0.0282
1.49263	-0.02075	1.53716	0.0282
1.55669	-0.02337	1.59984	0.03058
1.60154	-0.02473	1.61764	0.0339
1.69763	-0.02439	1.68028	0.03723
1.80013	-0.02549	1.734	0.03928
1.88981	-0.02557	1.8416	0.03976
1.95388	-0.02278	1.92243	0.03723
1.98591	-0.0205	1.95843	0.03422

1.99872	-0.01653	1.99445	0.0309
2.02434	-0.01281	2.04847	0.02599
2.04356	-0.00892	2.12025	0.02504
2.06919	-0.0052	2.20991	0.02583
2.06919	-9.72E-04	2.27261	0.02773
2.09481	0.00232	2.32631	0.03043
2.12684	0.0052	2.34412	0.03328
2.17809	0.00731	2.3709	0.03628
2.1909	0.01086	2.39772	0.03834
2.21653	0.01399	2.44249	0.04009
2.27418	0.01678	2.44238	0.04262
2.33184	0.01746	2.46022	0.04483
2.3959	0.01517	2.46909	0.04737
2.41512	0.01306	2.49586	0.05038
2.47918	0.01086	2.54062	0.05243
2.51121	0.00807	2.60328	0.05529
2.58168	0.00503	2.63003	0.05893
2.6073	0.00241	2.65672	0.06399
2.67136	-3.80E-04	2.71938	0.067
2.74183	2.11E-04	2.76412	0.06938
2.8123	0.00207	2.80886	0.07175
2.83152	0.00444	2.91643	0.07302
2.88277	0.00748	2.9704	0.06938
2.94042	0.01036	3.00638	0.06685
2.98527	0.01238	3.03342	0.06352
3.04933	0.01095	3.08737	0.06036
3.09417	0.00833	3.13234	0.05735
3.14542	0.00461	3.16836	0.05387
3.20307	0.00165	3.24917	0.05181
3.23511	-3.80E-04	3.28517	0.0488
3.31198	-0.00258	3.33013	0.04595
3.35682	-0.00402	3.47365	0.04548
3.42088	-0.00554	3.57223	0.04722
3.46573	-0.00714	3.69782	0.04643
3.49776	-0.00943	3.79655	0.04485
3.52338	-0.01196	3.86841	0.04216
3.59385	-0.01289	3.88647	0.03931
3.69635	-0.01416	3.89558	0.03599
3.78603	-0.01323	3.96741	0.03409
3.83728	-0.01179	3.98548	0.03092
3.86291	-0.00824	4.03944	0.02728
3.88213	-0.00469	4.11131	0.02459
3.92697	-0.00317	4.17405	0.02554
3.95259	8.88E-04	4.22774	0.02824

3.97822	0.00528	4.28147	0.03029
4.02947	0.00824	4.34415	0.03267
4.04869	0.01348	4.38884	0.03631
4.08712	0.01771	4.41558	0.04011
4.15119	0.0221	4.42436	0.0447
4.20884	0.02549	4.45111	0.04834
4.30493	0.02616	4.45099	0.05104
4.36259	0.02219	4.48672	0.05452
4.40102	0.01923	4.50452	0.05768
4.40102	0.01534	4.53125	0.0618
4.43306	0.01061	4.53111	0.06513
4.47149	0.00621	4.522	0.06845
4.49071	0.00334	4.52186	0.07193
4.54196	-6.34E-04	4.53066	0.07573
4.57399	-0.00283	4.57543	0.07763
4.60602	1.27E-04	4.58424	0.08143
4.64446	0.00317	4.62892	0.08523
4.67008	0.00731	4.71857	0.08603
4.70211	0.01086	4.78146	0.08349
4.73414	0.01433	4.84437	0.08033
4.77258	0.01847	4.87147	0.0759
4.81742	0.0216	4.94335	0.07273
4.8943	0.0221	4.97039	0.06956
4.99039	0.01991	5.02441	0.06466
5.04164	0.01737	5.02454	0.06149
5.08008	0.01298	5.06054	0.05864
5.12492	0.00959	5.12348	0.05484
5.16976	0.00604	5.1326	0.0512
5.2082	0.00309	5.15064	0.04883
5.26586	-8.03E-04	5.17774	0.04408
5.3107	-0.00359	5.2317	0.04076
5.39398	-0.00596	5.25871	0.03822
5.49007	-0.00748	5.34842	0.03759
5.56694	-0.0085	5.45595	0.03981
5.63101	-0.01103	5.57248	0.04123
5.74632	-0.01314	5.65315	0.04234
5.79757	-0.00959	5.68914	0.03981
5.81678	-0.00528	5.72514	0.03665
5.836	-0.00131	5.7522	0.03316
5.89366	0.00359	5.7972	0.02936
5.90647	0.00959	5.81527	0.0262
5.96413	0.01509	5.86024	0.02319
5.99616	0.01999	5.86934	0.02018
6.04741	0.02498	5.90534	0.01718

6.07944	0.02802	5.97714	0.01607
6.1435	0.02836	6.08472	0.01702
6.20115	0.02692	6.13845	0.01892
6.2524	0.02346	6.19216	0.0213
6.26521	0.02041	6.22788	0.02494
6.29725	0.01686	6.22773	0.02858
6.31006	0.01382	6.2545	0.03175
6.3549	0.01052	6.27227	0.0357
6.41896	0.00816	6.28999	0.04077
6.50865	0.00951	6.32566	0.04568
6.53427	0.01213	6.33443	0.05043
6.5599	0.01568	6.34319	0.05533
6.59833	0.01991	6.36987	0.06056
6.64318	0.02439	6.41451	0.06547
6.6688	0.02819	6.43228	0.06927
6.71364	0.03199	6.45001	0.07433
6.79693	0.0336	6.44085	0.07877
6.85458	0.03174	6.45866	0.08177
6.88661	0.03022	6.49438	0.08542
6.89942	0.02819	6.51216	0.08906
6.92505	0.0265	6.53893	0.09238
6.93786	0.02337	6.58361	0.09618
6.95708	0.02058	6.65529	0.09777
6.98911	0.01746	6.72718	0.0946
7.01473	0.01551	6.78112	0.09144
7.0788	0.01162	6.78126	0.08827
7.11723	0.009	6.83518	0.08558
7.16848	0.00647	6.86223	0.08225
7.20692	0.00359	6.87133	0.07909
7.23895	0.00123	6.8984	0.07513
7.28379	-0.00131	6.92547	0.07133
7.2966	-0.00393	6.93463	0.0669
7.35426	-0.0068	6.95278	0.06183
7.42473	-0.01061	6.99786	0.05613
7.48238	-0.01213	7.04027	0.05313
7.54004	-0.01306	7.02126	0.04815
7.65535	-0.00985	7.09822	0.04612
7.713	-0.00714	7.09838	0.04239
7.75144	-0.00292	7.11774	0.03899
7.77707	0.00173	7.15631	0.03594
7.83472	0.00672	7.17568	0.03221
7.86675	0.01095	7.23344	0.02995
7.9116	0.01441	7.29753	0.02916
7.92441	0.01813	7.36795	0.0304

7.95644	0.02092	7.3678	0.0338
8.03331	0.02447	7.44461	0.03527
8.10378	0.02625	7.50222	0.03651
8.18065	0.02354	7.57919	0.03414
8.19987	0.0205	7.63062	0.02984
8.21909	0.0172	7.68842	0.02645
8.23831	0.01348	7.70775	0.02374
8.26393	0.01137	7.75272	0.0208
8.27675	0.00782	7.76569	0.01706
8.29596	0.00503	7.81704	0.01469
8.39206	8.03E-04	7.87479	0.01254
8.44331	0.00376	7.9325	0.0113
8.48815	0.00672	8.07336	0.013
8.50737	0.0101	8.12453	0.01481
8.55221	0.01306	8.17564	0.0182
8.57143	0.01669	8.20751	0.02182
8.60987	0.02041	8.21373	0.02646
8.81486	0.02041	8.25196	0.03121
8.76361	0.02287	8.24534	0.03629
8.87252	0.01686	8.27722	0.03991
8.87892	0.01128	8.30274	0.04229
8.90455	0.0074	8.30256	0.04659
8.98142	0.00342	8.33446	0.04964
9.00064	7.19E-04	8.33427	0.05416
9.03267	-0.00249	8.35969	0.05902
9.09033	-0.00545	8.39164	0.06083
9.12236	-0.00816	8.39146	0.06502
9.19283	-0.01137	8.4298	0.06739
9.25048	-0.01441	8.45527	0.07101
9.29532	-0.0172	8.48077	0.07395
9.42345	-0.01906	8.53836	0.07554
9.50673	-0.01965	8.62166	0.07497
9.57079	-0.01475	8.65381	0.07226
9.62204	-0.01002	8.67319	0.06841
9.65407	-0.00664	8.71172	0.06627
9.6861	4.23E-05	8.76311	0.06287
9.73094	0.00731	8.78882	0.06095
9.76297	0.01145	8.79542	0.05643
9.81422	0.01585	8.83393	0.05462
9.8911	0.01982	8.8405	0.05066
9.94234	0.02109	8.87907	0.0475
10.0064	0.01703	8.90486	0.04377
10.0512	0.01399	8.94341	0.04105
10.0769	0.01019	8.98198	0.03777

10.1089	0.00537	9.02057	0.03416
10.1281	0.00156	9.02717	0.02963
10.1537	-0.00148	9.07852	0.02737
10.1858	-0.00461	9.12344	0.02545
10.2627	-0.00545	9.20674	0.02489
10.3139	-0.00207	9.26429	0.02749
10.3331	0.00309	9.277	0.02998
10.3844	0.0079	9.34096	0.03224
10.442	0.01298	9.36646	0.03529
10.4805	0.01619	9.45612	0.03574
10.5637	0.01762	9.52032	0.03269
10.6342	0.0156	9.55243	0.03077
10.6919	0.01095	9.59101	0.02727
10.7303	0.0063	9.61035	0.02455
10.7623	0.00283	9.64888	0.02218
10.8072	-0.00232	9.70025	0.01946
10.852	-0.00689	9.75156	0.018
10.9033	-0.0101	9.82213	0.01562
10.9481	-0.01331	9.92458	0.01675
11.0186	-0.01636	9.96292	0.01902
11.0762	-0.01864	10.0269	0.02196
11.1595	-0.02287	10.0587	0.02535
11.2812	-0.02659	10.0842	0.02953
11.3517	-0.02828	10.0968	0.0353
11.4414	-0.02751	10.1414	0.03949
11.499	-0.02329	10.1284	0.04412
11.5375	-0.01906	10.1794	0.04966
11.5631	-0.01289	10.1856	0.05498
11.5951	-0.00816	10.2175	0.0595
11.6272	-0.00199	10.2237	0.06402
11.6784	0.00435	10.2619	0.069
11.7233	0.00926	10.2745	0.07386
11.8386	0.01374	10.2871	0.07907
11.9347	0.01221	10.3253	0.08325
11.9923	0.00951	10.3316	0.08709
11.9987	0.00621	10.3764	0.08823
12.0372	0.00131	10.447	0.0854
12.0628	-0.00317	10.4983	0.08269
12.1204	-0.00655	10.5369	0.07929
12.1653	-0.00875	10.5755	0.07545
12.287	-0.00833	10.627	0.07104
12.3703	-0.00427	10.6656	0.06584
12.4407	-0.00207	10.7171	0.06007
12.5048	-0.00156	10.7365	0.05532

12.6009	-0.00368	10.7687	0.05103
12.6521	-0.00664	10.7753	0.04684
12.7226	-0.0101	10.814	0.04164
12.7611	-0.01247	10.8719	0.03452
12.8251	-0.015	10.8913	0.03124
12.8892	-0.01796	10.9747	0.02717
12.934	-0.01999	11.0324	0.02683
12.9725	-0.02227	11.0836	0.02887
13.0365	-0.02489	11.1412	0.03023
13.075	-0.02751	11.1796	0.03158
13.1262	-0.02946	11.2499	0.03407
13.2031	-0.03056	11.3011	0.03453
13.2992	-0.02997	11.3589	0.03193
13.3184	-0.02718	11.4231	0.02865
13.3568	-0.02337	11.4425	0.02571
13.376	-0.0205	11.4746	0.02265
13.4209	-0.01644	11.526	0.01847
13.4593	-0.01331	11.5711	0.01316
13.5106	-0.00816	11.6353	0.0109
13.5362	-0.00376	11.7313	0.01293
13.581	-2.11E-04	11.8273	0.01531
13.6643	0.00664	11.872	0.01734
13.722	0.00816	11.9232	0.01938
13.8053	0.00393	11.9807	0.02368
13.8373	0.00148	12.0317	0.02798
13.8693	-0.00173	12.0379	0.03273
13.8885	-0.00537	12.0826	0.03748
13.9078	-0.00934	12.14	0.04234
13.927	-0.01272	12.1527	0.04618
13.9846	-0.01669	12.2037	0.05071
14.0231	-0.015	12.2613	0.05229
14.0743	-0.01162	12.3703	0.0506
14.0935	-0.00731	12.4793	0.04868
14.132	4.23E-05	12.5114	0.04608
14.1704	0.00731	12.5692	0.0437
14.2345	0.01238	12.6141	0.04155
14.2601	0.01636	12.6335	0.03771
14.369	0.01822	12.6849	0.03409
14.4523	0.01407	12.7811	0.03115
14.5035	0.00943	12.8581	0.02957
14.5484	0.00545	12.935	0.02855
14.5932	0.00156	13.0118	0.02946
14.6252	-0.0019	13.0821	0.03308
14.6829	-0.0068	13.1076	0.03534

14.7341	-0.01095	13.178	0.03738
14.779	-0.01551	13.2549	0.03749
14.8559	-0.02041	13.3319	0.03342
14.9648	-0.02675	13.3577	0.02992
15.0352	-0.02895	13.4219	0.02619
15.1185	-0.0298	13.4605	0.02336
15.2402	-0.02506	13.4926	0.02065
15.2787	-0.01864	13.5248	0.01759
15.3107	-0.01399	13.5761	0.0159
15.3555	-0.00943	13.6402	0.01465
15.394	-0.00528	13.7234	0.01714
15.4324	-8.88E-04	13.7681	0.02054
15.4965	0.00655	13.8128	0.02449
15.5285	0.01052	13.8638	0.02981
15.6438	0.01331	13.8764	0.03524
15.7079	0.01188	13.9146	0.04146
15.7399	0.00858	13.9079	0.04711
15.7719	0.00495	13.9588	0.05491
15.804	-0.00173	13.965	0.06181
15.8296	-0.0074	13.9838	0.06962
15.8488	-0.01323	13.99	0.07516
15.9577	-0.01889	13.9834	0.08058
15.9962	-0.01517	14.0408	0.08567
16.0538	-0.01112	14.047	0.09054
16.0602	-0.00731	14.0917	0.09359
16.1307	-0.0019	14.1364	0.09687
16.214	-1.27E-04	14.2005	0.09585
16.3037	-0.00309	14.2583	0.0937
16.451	-0.00824	14.3225	0.08986
16.4894	-0.01162	14.3547	0.0859
16.5791	-0.0161	14.3869	0.08195
16.6432	-0.01788	14.4128	0.07663
16.6944	-0.02084	14.4513	0.07358
16.7393	-0.02396	14.4899	0.0694
16.9186	-0.02692	14.5093	0.06476
16.9635	-0.02997	14.5415	0.06159
17.034	-0.03056	14.5417	0.05718
17.13	-0.02329	14.5869	0.04904
17.1493	-0.0172	14.6448	0.04396
17.2005	-0.00959	14.6706	0.0383
17.2325	-0.00182	14.7092	0.03581
17.2838	0.00697	14.7478	0.03197
17.335	0.01306	14.8056	0.02688
17.4055	0.01948	14.8633	0.02587

17.4439	0.02278	14.9465	0.02869
17.5529	0.02354	14.9912	0.03118
17.6233	0.02008	15.1257	0.03243
17.6489	0.01534	15.2219	0.02971
17.6874	0.01078	15.2733	0.02655
17.7258	0.00528	15.3503	0.02463
17.745	-8.03E-04	15.4337	0.02169
17.7771	-0.00418	15.5426	0.02022
17.8155	-0.00773	15.6386	0.02361
17.8475	-0.00951	15.6897	0.02542
17.9052	-0.00571	15.7665	0.02904
17.9372	-0.00106	15.7983	0.03357
17.9885	0.00376	15.8237	0.03911
17.9949	0.00841	15.8427	0.04476
18.0461	0.01306	15.8873	0.05098
18.1294	0.01534	15.887	0.0563
18.1999	0.01407	15.9316	0.0632
18.2511	0.01205	15.925	0.06806
18.296	0.00892	15.9761	0.06998
18.3472	0.00545	16.008	0.07303
18.4177	0.00123	16.0271	0.07575
18.4689	-0.00123	16.1361	0.07507
18.533	-0.00393	16.1874	0.07258
18.5586	-0.00647	16.2324	0.06998
18.6035	-0.00858	16.2838	0.0658
18.738	-0.0112	16.3288	0.06184
18.7892	-0.01306	16.3803	0.05732
18.9045	-0.01458	16.4317	0.05348
18.943	-0.0134	16.4832	0.04805
18.9814	-0.01171	16.5218	0.04398
19.0263	-0.00875	16.6115	0.04206
19.0455	-0.00427	16.7589	0.04206
19.0839	-7.19E-04	16.8934	0.0424
19.116	0.00486	16.9316	0.04659
19.1544	0.01086	17.0085	0.04794
19.2056	0.0156	17.1302	0.0467
19.2185	0.01982	17.1752	0.04286
19.2697	0.02303	17.2138	0.03901
19.2889	0.02625	17.2908	0.03698
19.3466	0.02997	17.3613	0.03517
19.4042	0.03225	17.4383	0.03325
19.4683	0.03191	17.5601	0.03167
19.5195	0.02582	17.6368	0.03438
19.5324	0.02227	17.6943	0.03959

19.558	0.01796	17.7389	0.04434
19.5708	0.01433	17.7707	0.05022
19.6028	0.01112	17.8216	0.05949
19.6348	0.009	17.8341	0.06537
19.6733	0.00807	17.8466	0.07204
19.7181	0.01052	17.872	0.07883
19.7566	0.01391	17.9038	0.08324
19.7758	0.01805	17.9421	0.08606
19.8334	0.02278	17.9804	0.09025
19.8911	0.02912	18.0636	0.09161
19.9423	0.03183	18.1279	0.0872
19.968	0.03292	18.1793	0.08358
		18.2051	0.0786
		18.2501	0.07431
		18.2888	0.06933
		18.3338	0.06492
		18.3788	0.06074
		18.4367	0.05633
		18.4817	0.05226
		18.5459	0.0491
		18.6164	0.04774
		18.7251	0.0517
		18.7699	0.0534
		18.8339	0.05543
		18.9109	0.05294
		18.9814	0.05046
		19.02	0.04797
		19.0714	0.04492
		19.1163	0.04254
		19.1676	0.04051
		19.2062	0.03825
		19.3024	0.0352
		19.3473	0.03271
		19.4563	0.03203
		19.5009	0.03644
		19.5263	0.04176
		19.5582	0.04481
		19.5644	0.05001
		19.6027	0.0542
		19.6089	0.05974
		19.6471	0.0637
		19.6469	0.0689
		19.6787	0.07354
		19.6913	0.07874

19.7423	0.0853
19.7805	0.08993
19.883	0.09095
19.9088	0.08767
19.9474	0.08315
