# Supplementary material for: High resolution atomic force and Kelvin probe force microscopy image data of InAs(001) surface using frequency modulation method
Source: Data Brief. 2020 Jan 25;29:105177. doi: 10.1016/j.dib.2020.105177 (PMC7005429; doi:10.1016/j.dib.2020.105177)
Supplement: Multimedia component 3 [file mmc3.pdf]

X1 (tip-sample distance, nm)	Y1 (Δf_1, kHz)	X2 (tip-sample distance, nm)
-0.09145	-1.09172	-0.99435
-0.07301	-1.13542	-0.98299
-0.05173	-1.17388	-0.9759
-0.03185	-1.13724	-0.96867
-0.01188	-0.81413	-0.96016
0.0094	-0.84561	-0.95207
0.02785	-0.87009	-0.94186
0.04843	-0.88061	-0.93164
0.0683	-0.86667	-0.92824
0.08818	-0.84749	-0.9193
0.10806	-0.81259	-0.90653
0.12936	-0.78992	-0.89504
0.14853	-0.77249	-0.8844
0.16982	-0.75331	-0.86738
0.19041	-0.73413	-0.85163
0.211	-0.72369	-0.84269
0.23016	-0.71674	-0.82694
0.24933	-0.70629	-0.80991
0.27133	-0.6941	-0.79459
0.28979	-0.6854	-0.77756
0.30895	-0.66796	-0.76735
0.32954	-0.65752	-0.74989
0.34942	-0.64009	-0.73627
0.37	-0.6279	-0.72435
0.38917	-0.60872	-0.71328
0.41189	-0.59129	-0.70306
0.42964	-0.57385	-0.68731
0.45164	-0.54245	-0.67283
0.47081	-0.52502	-0.65921
0.48998	-0.51282	-0.63707
0.50986	-0.4919	-0.62174
0.52973	-0.47272	-0.60344
0.55103	-0.46402	-0.583
0.56949	-0.44659	-0.57491
0.59078	-0.42741	-0.55959
0.61066	-0.41347	-0.54767
0.63054	-0.3873	-0.5366
0.65113	-0.36463	-0.51148
0.67029	-0.35419	-0.48807
0.69088	-0.32977	-0.46039
0.71147	-0.31758	-0.44039
0.73205	-0.30539	-0.42123

0.75122	-0.29494	-0.40505
0.77039	-0.28274	-0.3927
0.79097	-0.27055	-0.37865
0.81084	-0.2671	-0.3646
0.83143	-0.26189	-0.34332
0.8513	-0.25319	-0.32203
0.87189	-0.24973	-0.29649
0.89034	-0.25326	-0.28074
		-0.25689
		-0.22837
		-0.20112
		-0.16919
		-0.13045
		-0.09127
		-0.07296
		-0.0538
		-0.02357
		-0.00867

Y2 (Δf_2, kHz)	X3 (tip-sample distance, nm)	Y3 (Δf_3, kHz)
-0.09435	-0.09097	-0.88997
-0.08299	-0.07079	-0.92006
-0.0759	-0.0506	-0.96192
-0.06867	-0.03095	-0.99462
-0.06016	-0.01077	-1.01948
-0.05207	0.00942	-1.02471
-0.04186	0.02907	-1.01817
-0.03164	0.04926	-1.01555
-0.02824	0.06944	-0.99986
-0.0193	0.08856	-0.96977
-0.00653	0.10928	-0.94099
0.00496	0.12946	-0.91352
0.0156	0.14911	-0.87689
0.03262	0.1693	-0.84157
0.04837	0.18948	-0.80756
0.05731	0.2102	-0.77747
0.07306	0.22985	-0.73823
0.09009	0.2495	-0.71206
0.10541	0.26916	-0.6859
0.12244	0.28775	-0.65843
0.13265	0.30899	-0.63488
0.15011	0.32918	-0.6048
0.16373	0.34936	-0.5734
0.17565	0.37008	-0.55247
0.18672	0.39026	-0.52762
0.19694	0.40832	-0.49491
0.21269	0.42957	-0.47791
0.22717	0.44975	-0.46352
0.24079	0.46834	-0.43605
0.26293	0.48853	-0.41642
0.27826	0.50977	-0.39549
0.29656	0.52836	-0.37849
0.317	0.55014	-0.36017
0.32509	0.56926	-0.34317
0.34041	0.59008	-0.32512
0.35233	0.61006	-0.30837
0.3634	0.6296	-0.29372
0.38852	0.65	-0.27907
0.41193	0.66955	-0.26547
0.43961	0.68909	-0.255
0.45961	0.70991	-0.24977
0.47877	0.73031	-0.24035

0.49495	0.74986	-0.23512
0.5073	0.76941	-0.22988
0.52135	0.7898	-0.22361
0.5354	0.81062	-0.21733
0.55668	0.82975	-0.21105
0.57797	0.84887	-0.20581
0.60351	0.87011	-0.20163
0.61926	0.88966	-0.20058
0.64311		
0.67163		
0.69888		
0.73081		
0.76955		
0.80873		
0.82704		
0.8462		
0.87643		
0.89133		
